# Prosecution of American Dentists

**Published:** 1888-09

**Authors:** 


### Editorial, Etc.

---

PROSECUTION OF AMERICAN DENTISTS.—At the Worship-street Police-court recently Dr. Huntley and Dr. Coe, summoned as of the American Dental Institute, 44, Finsbury square, appeared to answer a charge of having, on July 27, taken and used the letters "D. D. S.," or some other name, title, or description that they were registered under the Dentists Act, 1878, as specially qualified to practice dentistry, they not being registered under the said Act.—Mr. Melsheimer, barrister, supported the summonses (which by arrangement were taken together) and Mr. Waddy, Q. C., defended.—Mr. Melsheimer, said that the prosecution was taken under the Act 41 and 42 Vic., and the prosecutors were the British Dental Association. In the pamphlet issued by the American Dental Institute the names of defendants appeared, followed by the letters "D. D. S. Pennsylvania University," and "D. D. S., Boston College," respectively. Neither of these qualifications was recognised by the General Council of the British Dental Association, which only recognised degrees conferred by Harvard and Michigan Universities (U. S. A). --Mr. Thomas Smith, solicitor's clerk, said he was employed by Messrs. Beaumont and Bouverie, solicitors to the British Dental Association. On July 27, he, acting under instructions, went to the American Dental Institute, 44, Finsbury-square, where, in a waiting room, his name and address were taken by a lady, who seemed to act as secretary. She handed him a pamphlet of the institute, on the inner first page of which the names of the defendants, among others appeared. After that he was shown into the operating room. There he saw the defendant Huntley, to whom he said, "Dr. Huntley, I presume?" The defendant answered "Yes," and witness then pointed out the name of Huntley, asking if it was his, and what the letters "D. D. S." meant. The defendant replied, "They mean doctor of dental surgery." Witness then asked if the pamphlet was issued with his (Huntley's) authority, and the defendant then wished to know why the questions were put, and witness told him. Mr. Huntley then said that his name was published with the letters with his consent. In a following conversation the defendant said that the principal of the institute was Dr. Clifford, who was registered, but that he (Huntley) knew that nearly all the others were not. He added that the British Association could not touch them, as they worked for Dr. Clifford. Witness afterwards saw the defendant Coe.—Mr. Melsheimer then put in the list of registered dentists, to show that the defendants' names did not appear therein, and in re-examination the witness said that the defendant Huntley said that though he was qualified every impediment was placed in the way of Americans practicing in this country.—Mr. Waddy, for the defence, admitted that the defendants were not registered, but said they could not be, though duly qualified. He thought the Act a most mischievous one, and that the exclusion of such colleges as those from which the defendants received their diplomas was never intended to have the effect of preventing them practicing as qualified persons in this country. If the British Medical Council would recognise that fact there would be more sense than in their playing the part of the three tailors of Tooley-street. Mr. Waddy then proceeded to argue that the defendants had not offended against the legal intention of the Act by announcing themselves as "D. D. S.," because that was not a recognised "special qualification," and he said that it would be as stupid to take it as meaning that as it would be to assume that a gentleman entitled to put "D. D." after his name announced himself as a capable theologian, or that one writing "M. A.," often a purely honorary degree, after his name was capable of editing a Greek play.—Mr. Bushby reserved his decision.

Mr. Bushby, the sitting magistrate at Worship-street Police Court, London, on Tuesday, delivered judgment in the prosecution of Messrs. Coe and Hunter, summoned as "doctors" of the American Dental Association, 44 Finsbury square, for having within the past six months taken and used the letters "D. D. S.," or some other name, title, or description, implying that they were registered under the Dentists Act, 1878, or were specially qualified to practice dentistry, they not being registered, &c. Mr Bushby said—The question here is whether the defendants, by describing themselves as "doctors of dental surgery" of Pennsylvania University and Boston College respectively, have used titles implying that they were registered under the Dentists Act, 1878, or that they were specially qualified to practice dentistry. In either case since they were not in fact registered, they would be liable under the 3rd section to a penalty of £20. The act seeks to protect the public against quacks and knaves by the following amongst other provisions:—"Persons styling themselves dentists must be on the register, and are liable to be struck off for misconduct. Before being registered they must satisfy the general registrar that they have proper certificates of competency, and only such foreign certificates are admissible for the purpose as are recognised in the list published by the general council." Now, certificates from Pennsylvania University and Boston College are not included in this list. I think, therefore, that the defendants cannot be said to have used titles which implied registration, and the only remaining point is whether the titles imply a special qualification to practice dentistry. It was urged for the defence that the words "specially qualified" must be restricted to recognised qualifications, such as D.

D. S. of Michigan or D. D. M. of Harvard. But this would go far to cripple the Act, for the only titles verbally specified in the 3rd section are "dentist" and "dental practitioner." By merely substituting such equivalents as dental surgeon or doctor of dentistry, or, as the defendants done here, doctor of dental surgery, the protection to the public would be slight indeed. On the other hand, if the Legislature meant to enforce registration on everyone using a title which implied that he was specially qualified to practice dentistry in any way whatever, that person would come within the Act as effectually as if he styled himself a "dentist" or "dental practitioner." The latter seems to me the rational view, and I therefore convict the defendants. Mr. Waddy has dwelt on the hardships of their particular diplomas being unrecognised as titles to registration. But, before practicing here, I do not see why they should not have qualified by the means open to English dentists. Taking into consideration, however, that the clause might have been more clearly expressed—and no question has been raised as to the right of the defendants to the designations they have adopted, I think it will be sufficient to impose in each case a fine of £5 and 2s costs.

The Solicitor for the defendants intimated that he would give notice of appeal.

[This discrimination against reputable American Dental Schools on account of one year's longer attendance, is simply ridiculous, and the Judge who tried the case appears to be of this opinion by the lightness of the fine he could not help imposing. With all due respect to the two American Schools recognized in England merely on account of a three year's course, we venture to say that either the University of Pennsylvania Dental Department, or the Boston Dental College gives more practical and equal theoretical instruction, than either of the two American Schools placed among those recognized in England.—EDITOR.]

